# Ameliorative Effects of Lycopene and L‐Carnitine on CCl_4_‐Induced Liver Fibrosis Rat Model

**DOI:** 10.1002/fsn3.71689

**Published:** 2026-04-10

**Authors:** Adeel Shahid, Somia Shehzadi, Moazzam Salamat, Sahar Shafiq, Zaid Talal Abdulqader Ali

**Affiliations:** ^1^ University Institute of Medical Laboratory Technology The University of Lahore Lahore Pakistan; ^2^ Faculty of Medicine University of Saba Region Marib Yemen

**Keywords:** inflammation, L‐carnitine, liver fibrosis, lycopene, oxidative stress

## Abstract

Liver toxicity is a major health concern caused by pharmaceutical exposure, poisons like CCl4, or environmental contaminants. The CCl4‐induced liver toxicity model is extensively used to study hepatic damage, such as oxidative stress and fibrosis. In current study, synergistic effect of natural compounds Lycopene (Lyc) and L‐Carnitine (L‐Car) possessing the antioxidant activity was assessed to mitigate the liver fibrosis induced by CCl4 in male rat model. CCl4 treated rats showed significant decrease in body weight (21.62% ± 0.83%) alongside elevated liver enzymes ALP (276 ± 6.62), AST (283 ± 4.53), ALT (138 ± 0.74), bilirubin (1.73 ± 0.74) and lactate dehydrogenase (LDH) an injury marker (0.778% ± 0.06%). After treatment of CCl4 induced fibrosis with Lyc + L‐Car, significantly increased body weight of rats was observed (34.39% ± 0.77%). Liver enzymes also showed remarkable improvement after treatment with Lyc + L‐Car (*p* ≤ 0.001). Combined Lyc + L‐Car group showed reduced the LDH level (0.246% ± 0.02%), fibrosis gene markers TIMP‐1 and Col1α1 (*p* ≤ 0.001) and increased antioxidant enzyme activity of SOD (0.56 ± 0.04 U/dL) and CAT (0.489 ± 0.004 U/dL). Histological analysis showed a marked improvement in liver architecture, with reduced fibrosis appearance. These findings suggest that combination of Lyc and L‐Car supplementation effectively counteracts fibrosis, oxidative stress, and liver enzymes elevation, supporting its potential role as a dietary therapeutic agent for metabolic and hepatic disorders. Future recommendations include conducting long‐ term clinical trials in humans to validate these findings, exploring optimal dosages for dietary lycopene supplementation, and investigating its molecular mechanisms of action.

## Graphical abstract

Rats with CCl_4_‐induced liver fibrosis may benefit from the synergistic antifibrotic effects of lycopene (30 mg/kg) and L‐carnitine (200 mg/kg). Treatment improves liver function tests and lowers oxidative stress, hepatic fibrosis, LDH levels, and body weight loss.

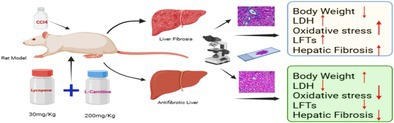

## 1 Introduction

The liver is one of the most important glandular organs for chemical and energy metabolism and barrier activities making up 2% of an adult's body weight (Jia et al. 2024). It produces hormones, stores glucose, regulates digestive system absorption, and delivers these chemicals to the blood (Andrews et al. 2024). Environmental contaminants, industrial chemicals, drugs, alcohol, metabolic syndrome, obesity, diabetes, and abnormal liver enzyme levels can cause fibrosis, cirrhosis, liver failure, and cancer (Åberg et al. 2025). Due to its high metabolic activity and extensive blood supply, the liver is constantly exposed to oxidative damage, inflammation, fibrosis, and cirrhosis (Tiwari et al. 2025). The Global Burden of Disease 2019 report says liver illnesses kill over one million people annually and cirrhosis killed one million people worldwide (Wang, Wang, et al. 2024). Geographic disparities in risk factor distribution and healthcare accessibility are also increasing advanced fibrosis and cirrhosis worldwide (Zamani et al. 2025).

Carbon tetrachloride (CCl4), commonly used as an experimental hepatotoxin, mimics human liver issues such as oxidative stress, inflammation, apoptosis, fibrosis, and cirrhosis. CYP2E1 cytochrome P450 enzymes create damaging reactive free radicals such as –CCl_3_ and –OOCCl_3_ during CCl_4_ metabolism. Radical lipid peroxidation damages hepatocyte membranes, ER, and mitochondria. It causes necrosis and apoptosis. ATP production drops and pro‐apoptotic substances deplete hepatocytes due to mitochondrial malfunction (Mrwad et al. 2025).

CCl_4_ degrades polyunsaturated fatty acids, producing lipid peroxidation, cell damage, and oxidative stress. Oxidative damage depletes glutathione (GSH) and antioxidant enzymes like superoxide dismutase (SOD), weakening cells and worsening damage. Oxidative stress triggers Kupffer cells to release pro‐inflammatory cytokines such as TNF‐α, IL‐6, and TGF‐β1. These cytokines stimulate hepatic stellate cells and cause inflammation and fibrosis (Li et al. 2023). Drugs to treat liver fibrosis or boost regeneration remain elusive despite intensive study. Antioxidant and anti‐inflammatory drugs provide benefits, but their long‐term efficacy and side effects limit their use. Natural compounds with multiple biological effects that reduce oxidative stress, inflammation, and fibrosis must be studied (Ibrahim et al. 2022).

Lycopene is a natural bioactive compound found in red foods such as tomatoes, and helps to combat free radicals. It reduces lipid peroxidation and strengthens membranes by inhibiting singlet and reactive oxygen species. Studies show that lycopene boosts liver cell's SOD and catalase (CAT) activities. This protects cells from oxidative damage from toxins like CCl4 (Kushwah et al. 2025). In addition to antioxidant benefits, lycopene lowers inflammation by inhibiting NF‐κB activation and reducing TNF‐α and IL‐6. In experimental and clinical investigations, lycopene protects against fatty liver disease and chemically induced hepatic damage (Ahn et al. 2012).

L‐carnitine boosts hepatocyte energy production via promoting β‐oxidation of long‐chain fatty acids in mitochondria. When CCl4 toxins harm the liver, mitochondrial malfunction and insufficient β‐oxidation lead to lipid buildup and cell death. L‐carnitine supplementation enhances mitochondrial activity, boosts β‐oxidation, and lowers oxidative stress and liver enzyme problems (Oh et al. 2022). Clinical and animal research shows L‐carnitine's hepatoprotective effects, improving histology and liver function assessments and safety (Li et al. 2021).

Despite research on lycopene and L‐carnitine, there is a lack in data on their combined effectiveness in preventing CCl4‐induced liver damage. This study examines their individual and synergistic hepatoprotective effects in a rat model, focusing on biochemical liver function, oxidative stress markers, histological changes, and molecular analysis of fibrosis gene markers. The findings may help create new liver disease treatments using these molecules.

## 2 Materials and Methods

### 2.1 Ethical Approval for Animal Model

Male Sprague–Dawley rats weighing 180–200 g and aged 6–8 weeks were acquired from the animal house of The University of Lahore in Pakistan. They were separated into four groups of eight (*n* = 8) and kept in a hygienic, well‐ventilated environment with a 12‐h light/dark cycle (25°C–27°C). The rats had access to running tap water and were fed according to a regular schedule. For one week before the experiment, they were acclimated in a lab setting; they were given water and a basic diet consisting of 5% fat, 65% carbohydrates, 20% protein, 5% fiber, 4% salt combination, and 1% vitamins.

Departmental Research Ethical Committee of The University of Lahore authorized the procedures used, guaranteeing adherence to animal care regulations (UOL/DERC/22/08/7524). The study minimized animal suffering by adhering to humanitarian principles.

### 2.2 Induction of Liver Fibrosis in Rats Using Carbon Tetrachloride (CCl_4_ )

Liver fibrosis was chemically induced in rats through intraperitoneal injections of CCl_4_. A solution of CCl_4_ (2 mL/kg body weight, 50% v/v in olive oil) was administered once weekly for four consecutive weeks.

### 2.3 Preparation and Administration of Lycopene and L‐Carnitine Solutions

Lycopene and L‐carnitine (L‐Car) were procured from a local pharmacy in Lahore, Pakistan, and stored at room temperature until use. Lycopene solution was prepared by dissolving in sterile saline and administered orally by gavage after induction of liver fibrosis, at a dose of 30 mg/kg body weight daily for 4 weeks (Noreen et al. 2025; Huang et al. 2022). L‐Car was similarly dissolved in sterile saline and administered orally at a dose of 200 mg/kg body weight once daily for 4 weeks following the induction of liver fibrosis using CCl_4_ (Andrews et al. 2024; Murata et al. 2022; Mollica et al. 2020).

### 2.4 Experimental Design

In total 40 rats as an animal model were split into five groups (eight rats per group) following a week of adaptation: Normal liver of rats (*n* = 8) without any injury or treatment was used as a negative control (−ve control), whereas rats injected with CCl4 intraperitoneally were used as positive control (+ve control). After four weeks, the +ve control group was further split up into three groups: CCl4 + Lycopene, CCl4 + L‐Carnitine, CCl4 + Lycopene+L‐Carnitine (Table 1). Lycopene (30 mg/kg b.w.), L‐Car (200 mg/kg b.w.), and a combination of lycopene (30 mg/kg b.w.) and L‐Car (200 mg/kg b.w.) were administered to the CCl4 groups. Rats' body weights (g) were also noted for each group (Noreen et al. 2023).

**TABLE 1:** Animal model groups.

Groups	*n*	Control and liver cancer rats	Treatment	Duration
−ve	8	Healthy control	Basal or normal diet	4 weeks
+ve	8	CCl4 treated	Basal or normal diet	Once a week (for 4 weeks)
	8	CCl4 + Lycopene	Lycopene (30 mg/kg b.w)	4 weeks
	8	CCl4 + L‐Carnitine	L‐Carnitine (200 mg/kg b.w)	4 weeks
	8	CCl4 + Lycopene+L‐Carnitine	Lycopene (30 mg/kg b.w) + L‐Carnitine (200 mg/kg b.w)	4 weeks

### 2.5 Liver Function Assay

Hematological parameters were performed by collecting the blood in anticoagulant tubes. At room temperature, the samples were allowed to coagulate for half an hour. To extract the serum, the samples were centrifuged at 500 g for 10 min. To evaluate liver function, the levels of bilirubin, alkaline phosphatase (ALP: Bioassay System, USA), alanine aminotransferase (ALT: DiaSys, Germany), and aspartate aminotransferase (AST: DiaSys, Germany) in the serum were measured following the protocol mentioned in the kit. Each reaction mixture's absorbance at specific wavelengths (ALP, ALT, and AST at 450 nm whereas ALT at 510 nm) was measured using a spectrophotometer, and the enzyme activity was reported in units per liter (U/L) (Noor et al. 2025; Baig et al. 2021).

### 2.6 Assessment of Antioxidant Enzyme Activity

Antioxidants SOD and catalase (CAT) activities were measured by following a recently reported method to assess the antioxidant response level of blood samples. The absorbance of SOD was measured at 560 nm, and activity was expressed in units per decilitre (U/dL). The absorbance of CAT was taken at 240 nm using a ultraviolet (UV) visible spectrophotometer. The CAT activity was expressed in units per gram (U/g) of tissue (Barakat et al. 2022).

### 2.7 Assessment of Lactate Dehydrogenase Assay (LDH)

The serum taken from the blood of each experimental group was used to measure the liver's LDH activity in accordance with the manufacturer's instructions (AMP Diagnostics, Austria). The LDH assay involved combining 5 μL of blood serum from each experimental group with 100 μL of working reagent in a 96‐well plate and then incubating the mixture for 5 min. Absorbance at 340 nm was measured using a spectrophotometer.

### 2.8 Gene Expression Analysis by qPCR

The mRNA expressions of several liver physiologic markers, including Col1α1 and Timp‐1, were assessed by qPCR (Elzoheiry et al. 2022). Primers along with sequences are presented in Table 2.

**TABLE 2:** Primer sequences used in real time‐PCR (Baig et al. 2021).

Gene	Forward primer	Reverse primer
TIMP‐1	TCCCCAGAAATCATCGAGAC	TCAGATTATGCCAGGGAACC
Col1α1	CAAGATGGTGGCCGTTACTAC	TTAGTCCTTACCGCTCTTCCAG

### 2.9 Histopathology of Liver

As is standard procedure, liver sections were fixed for 24 h in formaldehyde buffer (10%), then further sectioned into 5 μm pieces using a microtome and lodged in paraffin wax. Hematoxylin and eosin (H&E) staining was used to conduct routine histological investigation on the sections. For assessment of fibrotic area, sections were stained with Masson's Trichome (Richard Allen Scientific, USA) as per manufacturer's protocol. All sections were examined using an Olympus BX‐50 light microscope set to 200× magnification to assess the degree of hepatic injury or damage, and pictures were taken (Li et al. 2017).

### 2.10 Statistical Analysis

All statistical analysis was performed by using Graphpad Prism. One‐way and two‐way ANOVA tests were used to evaluate differences between control and treatment groups following Bonferroni post hoc test; *p* ≤ 0.05 was considered to be significant.

## 3 Results

### 3.1 Effect of Lycopene and L‐Carnitine on CCl4 Injured Rat Groups

On following 2 weeks of a CCl4 in rats, the CCl4 group's body weights (b.w) (21.62% ± 0.83%) were considerably lower than those of the control group (37.00% ± 0.77%). Therefore, Lycopene‐30 mg/kg (31.83% ± 0.86%) and L‐Car 30 mg/kg (32.43% ± 0.74%) improved the weight of the rats comparable to the untreated (UT) group (37.00% ± 0.77%). But the group given combined Lyc + L‐Car showed a significant (*p* ≤ 0.03) increase in b.w (34.39% ± 0.77%) (Table 3).

**TABLE 3:** Impact of CCl_4_‐damaged rats' livers treated with lycopene (Lyc) and L‐carnitine (L‐Car) on body weight to relative weight of liver (%), body weight growth percentage, and total liver weight (g).

Treatment	b.w growth %	Total liver weight (g)	b.w to relative weight of liver %	Total body weight
Control	37.00 ± 0.77++	8.10 ± 0.33++	0.069 ± 0.03++	372 ± 10.52++
CCl4	21.62 ± 0.83**	7.62 ± 0.89**	0.05 ± 0.01**	276 ± 11.37**
Lyc‐CCl4	31.83 ± 0.86++	7.69 ± 0.15++	0.066 ± 0.02++	301 ± 12.68++
L‐Car‐CCl4	32.43 ± 0.74++	7.61 ± 0.16++	0.063 ± 0.02++	296 ± 9.24++
Lyc + L‐Car‐CCl4	34.39 ± 0.77++	7.78 ± 0.16++	0.069 ± 0.01++	359 ± 13.75++

*Note:* Mean ± SEM (*n* = 8).

** Significance from the control group (normal) at *p <* 0.01.

^++^ Significance from the CCl4 injury group at *p <* 0.01.

### 3.2 Assessment of Liver Enzymes

**FIGURE 1:**
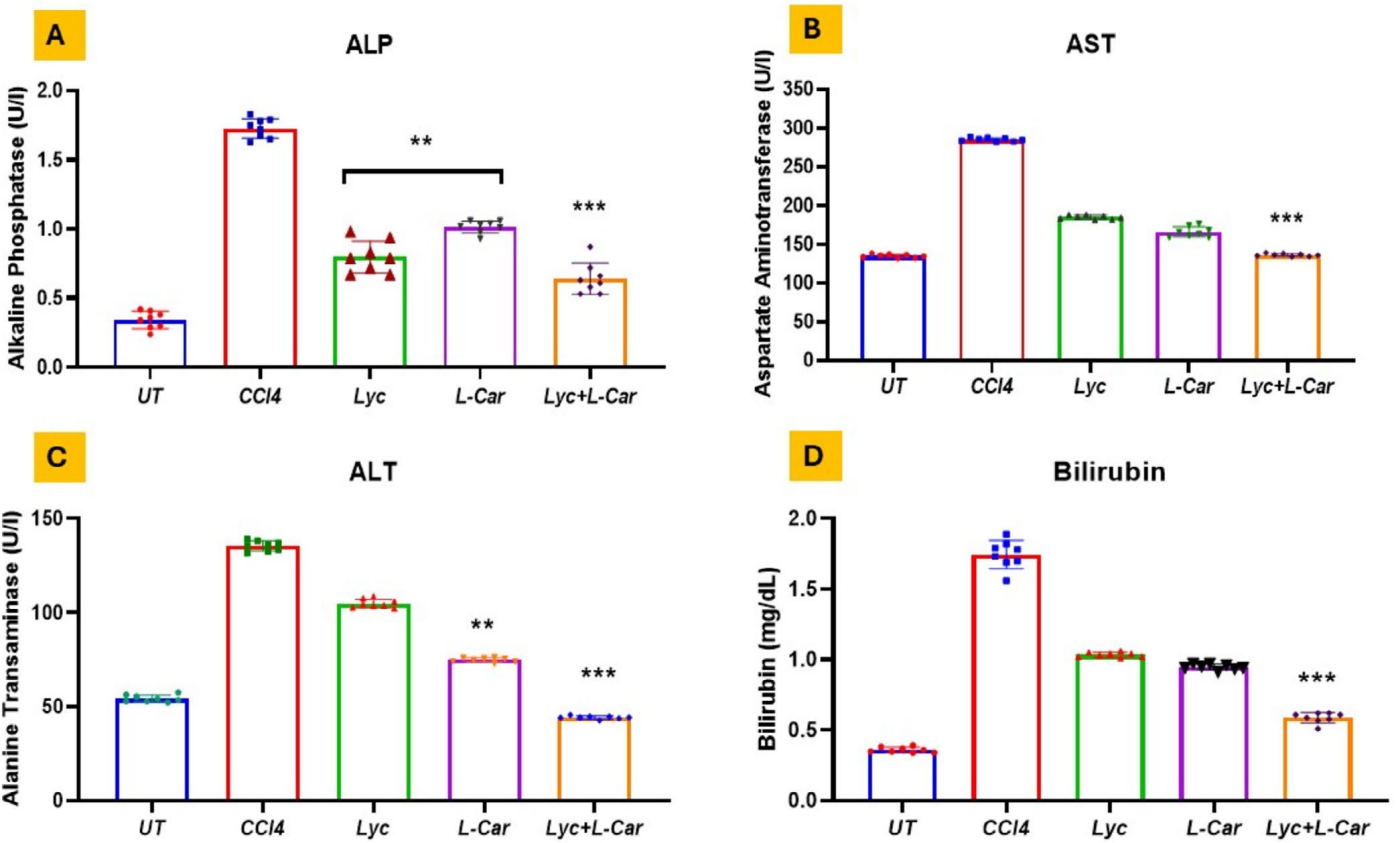
Assessment of liver enzymes of rat model with CCl_4_ injury. Mean ± SEM **p < 0.01, and ***p < 0.001 were used to express the data.

When compared to the control group (Group 1), rats administered CCl4 (Group 2), Lyc (Group 3), L‐Car (Group 4), and Lyc + L‐Car (Group 5) showed significant liver damage. Following a CCl4 injection, rats' blood levels of many liver enzymes, such as bilirubin (1.73 ± 0.74), ALT (138 ± 2.24), ALP (276 ± 6.62), and AST (283 ± 4.53), dramatically increased. Rats given Lyc (Group 3), L‐Car (Group 4), or Lyc + L‐Car (Group 5) had lower liver enzyme levels (Figure 1A–C) and bilirubin (Figure 1D) than the CCl4 group (Group 2). All Lyc + L‐Car groups showed a significant decrease in liver enzymes (***p < 0.001) (Figure 1).

### 3.3 Effect of Lycopene on the Antioxidants SOD and CAT Activity

**FIGURE 2:**
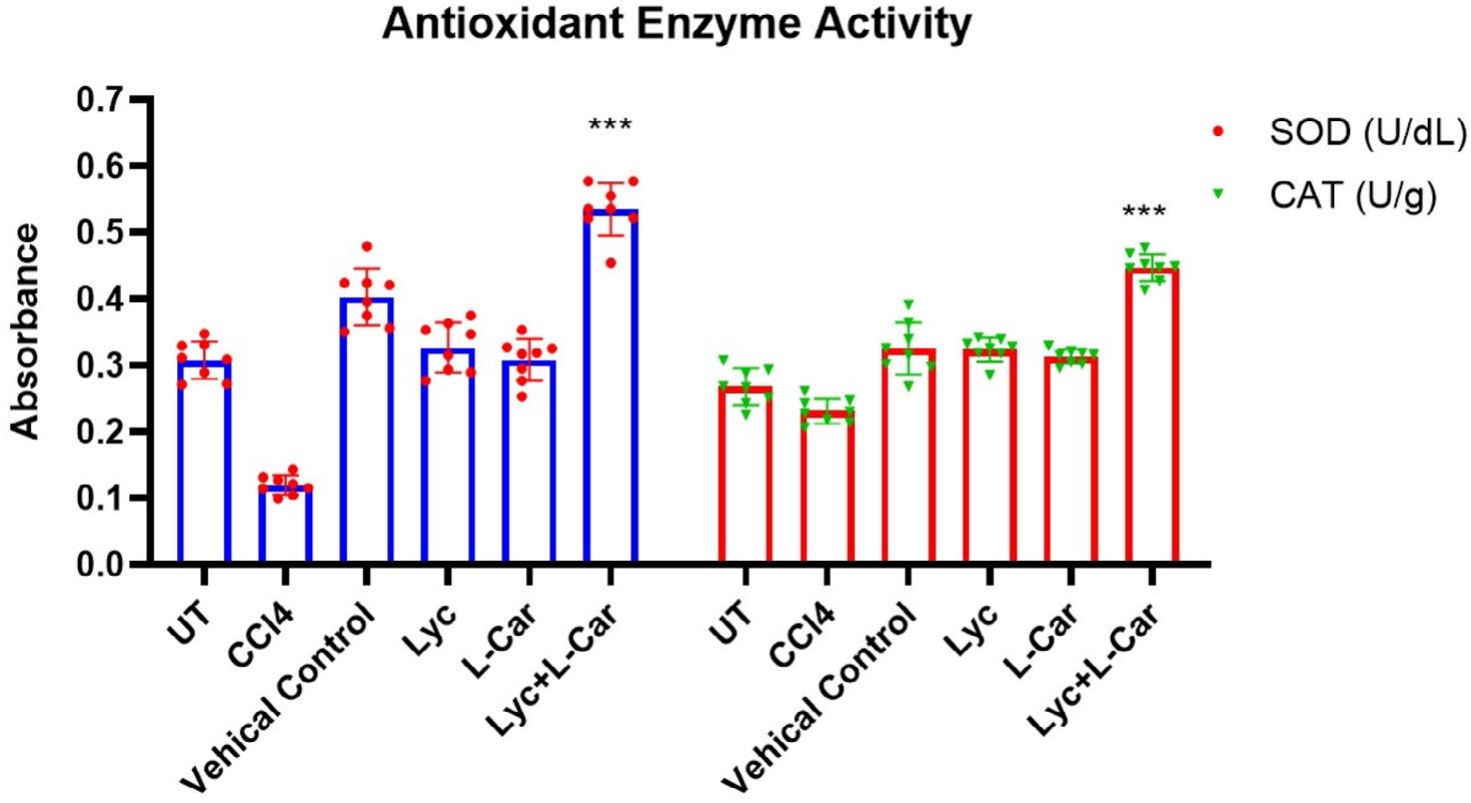
Lyc + L‐Car impact on the SOD (in blue) and CAT (in red) activities in the liver tissues of rats injured with CCl_4_. Mean ± SEM ***p < 0.001 was used to express the data.

In our study the −ve control group had an increased SOD activity in rat serum (0.347 ± 0.04 U/dL) than the +ve control group (0.21.12 ± 1.22 U/dL) (*p* ≤ 0.05). Additionally, compared to the −ve control rats (0.277 ± 0.05 U/g), the CAT enzyme activity was significantly reduced in the CCl4 group (0.153 ± 0.06 U/g, respectively). However, the Lyc + L‐Car group had considerably increased CAT enzyme (0.489 ± 0.05 U/dL, respectively) and SOD (0.56 ± 0.04 U/dL) activity contrary to the CCl4 group (Figure 2).

### 3.4 Evaluation of Lactate Dehydrogenase Release

**FIGURE 3:**
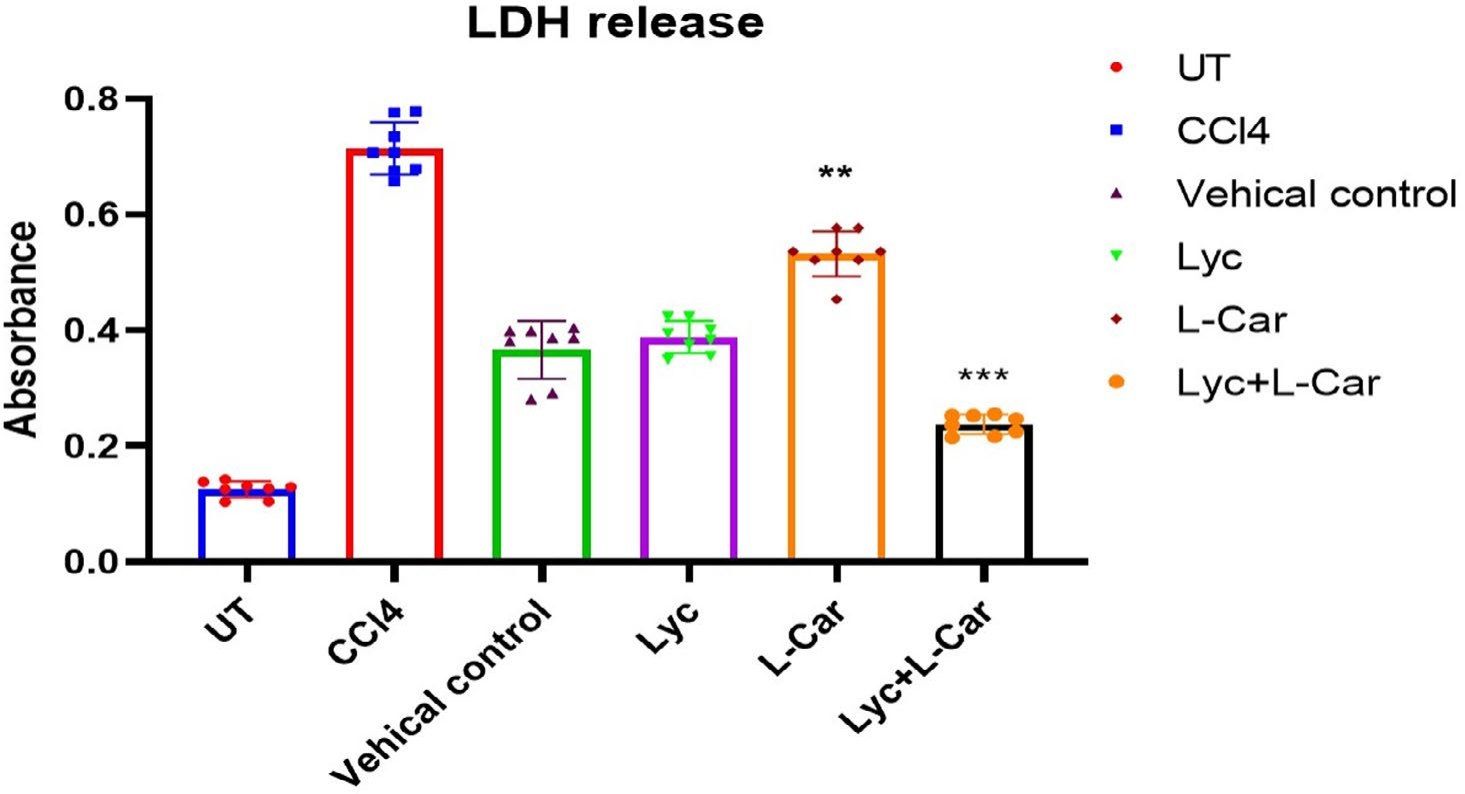
Evaluation of lactate dehydrogenase release in liver damage models that are −ve (UT) and +ve (CCl_4_). Graphpad Prism software was used to express all data as mean ± SEM, with **p < 0.05 and ***p < 0.01.

**FIGURE 4:**
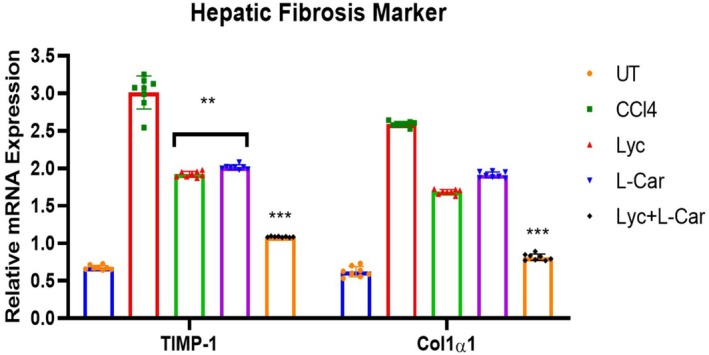
Assessment of fibrosis gene markers by real time‐PCR: Col1α1 and TIMP‐1, normalized to β‐Actin and expressed as the fold change versus the control. Data was expressed as Mean ± SEM (*n* = 8). **p ≤ 0.01, ***p ≤ 0.001 vs. CCl4 group.

To measure the amount of the cytoplasmic enzyme lactate dehydrogenase (LDH), which is released from the cell's cytosol when it is injured or under stress, an assay was conducted. According to Figure 3, the CCl4‐treated group had a greater level of LDH release (0.778% ± 0.06%) than the UT group (0.086% ± 0.02%). Compared to the CCl4, the Lyc + L‐Car group exhibited more significant results (0.246% ± 0.02%), indicating that the combination of Lyc and L‐Car helps to lower the injury level in liver fibrosis (Figure 4).

### 3.5 Gene Expression Analysis

Using molecular analysis, the impact of combined Lyc and L‐Car treatment on the control of several hepatocyte fibrosis indicators was investigated. To quantify the quantities of mRNA, semiquantitative PCR (qPCR) was employed. We found that the fibrotic markers Col1α1 had higher mRNA expressions in the CCl4 group (2.68 ± 0.56‐fold) than in the control group. Lyc + L‐Car therapy, on the other hand, dramatically reduced Col1α1 levels as near to normal control as feasible (Col1α1: 0.65 ± 0.04‐fold against control; 0.876 ± 0.046). The gene level of tissue inhibitors of matrix metalloproteinase‐1 (TIMP‐1) was evaluated to confirm the antifibrotic effects of Lyc + L‐Car on liver tissue damaged by CCl4. The CCl4 group's Timp‐1 mRNA expressions were roughly 4.13 ± 0.68 times greater than the UT group's (0.548 ± 0.03 times). But as expected, as Figure 4 illustrates, the fold increase was more drastically inverted in the Lyc + L‐Car group (TIMP‐1: 1.082 ± 0.35‐fold versus control group) than in the Lyc (TIMP‐1: 1.92 ± 0.28‐fold versus control group) and L‐Car groups (2.035 ± 0.36‐fold versus control).

### 3.6 Histological Analysis of Liver Sections

**FIGURE 5:**
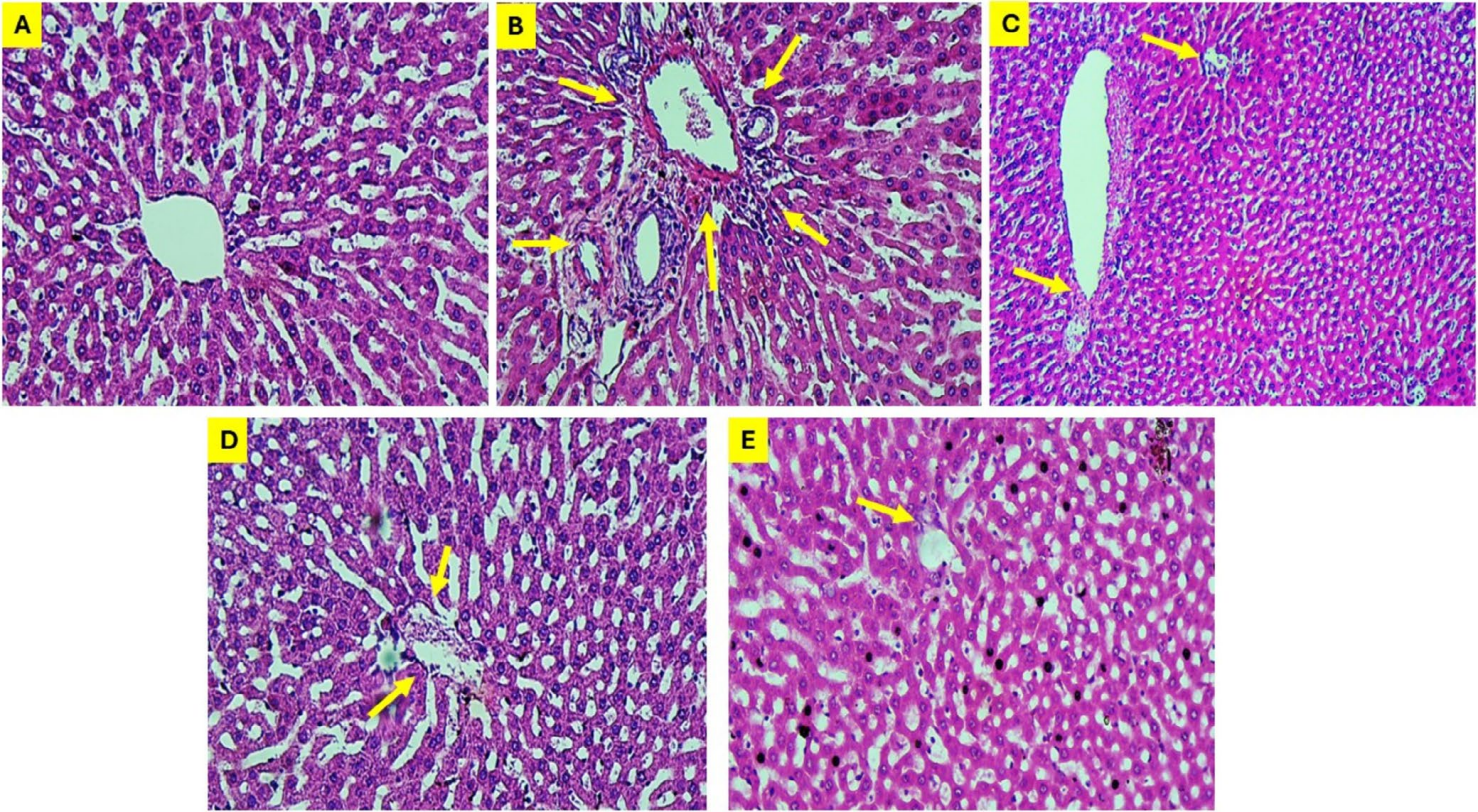
Rat liver tissue sections obtained from different experimental groups. The control group exhibited normal histological features of the liver, with no administered injuries, and the central vein appeared normal. (A) The liver of group1 rats were untreated and used as −ve control, group 2 rats administered CCl_4_, as shown in (B), exhibits notable degenerative changes, including altered histological architecture, vacuolization (circle), triaditis, and obstructed portal tract vasculature. (C) presents liver sections affected by CCl_4_ that have undergone treatment with Lyc (group 3). The central vein (CV) exhibits partial obstruction, and the sinusoids are observed to be dilated (yellow arrow). (D) Group 4 exhibited enhanced hepatic architecture characterized by moderate congestion and inflammatory hepatocytes following treatment with CCl_4_ and L‐Car. (E) Following administration of CCl_4_ and a combination of Lyc and L‐Car, the hepatic morphology of group 5 closely resembled that of the control group (group 1), exhibiting only a limited presence of inflammatory hepatocytes (yellow arrow) (H&E ×200).

**FIGURE 6:**
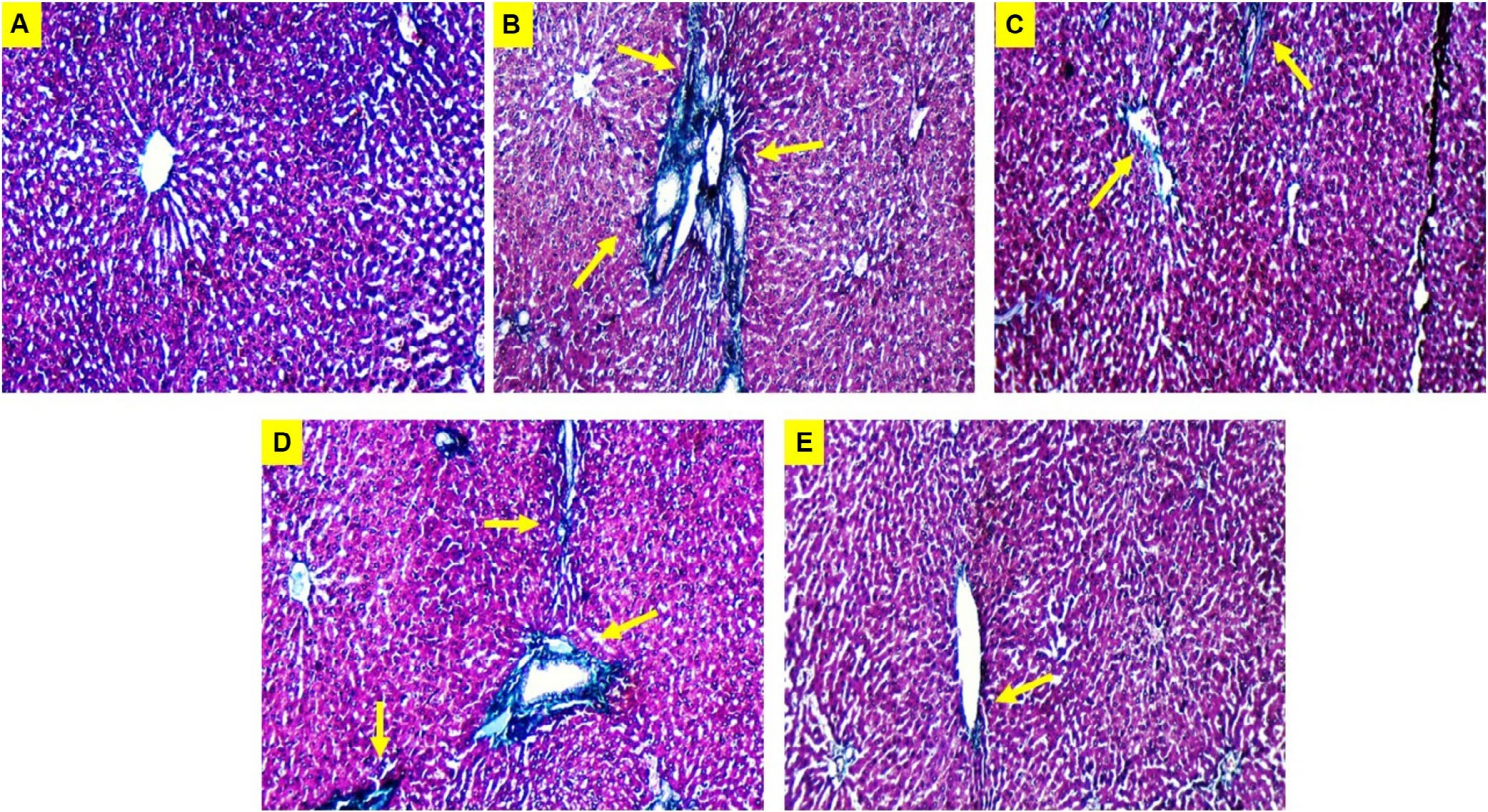
Characterization of fibrosis and collagen deposition in treated and untreated liver tissue sections. Appearance of the blue stained area shows the massive deposition of collagen (yellow arrow). Images were analyzed using Image J software (200×).

Rats administered with CCl4 displayed significant degenerative alterations, characterized by distorted histological architecture, vacuolization, severely congested central veins, congested portal tract vasculature, extensive inflammatory cell infiltration, and both congested and dilated sinusoids, as evidenced by liver sections stained with H&E. The hepatic architecture showed improvement in CCl4 rats treated with Lyc (Figure 5C), L‐Car (Figure 5D), Lyc + L‐Car (Figure 5E); however, moderate congestion between the hepatocytes remained evident (yellow arrow). The liver tissues of uninjured rats exhibited normal morphology, characterized by a central vein surrounded by hepatocytes (Figure 5A). Masson's Trichome staining further confirmed the results of the combination of Lyc + L‐Car in reducing fibrosis (Figure 6A–E).

## 4 Discussion

Lycopene is a fat‐soluble carotenoid pigment giving red color to tomatoes, characterized by significant antioxidant, anti‐apoptotic, and anti‐inflammatory properties, and it plays a role in the modulation of essential metabolic processes within the body (Zaidi 2023; Albrahim and Alonazi 2021). An amino acid derivative called L‐carnitine is necessary for energy metabolism; a lack of it can impair vital liver metabolic functions such as fatty acid metabolism, albumin formation, ammonia reduction through the urea cycle, and gluconeogenesis (Musazadeh et al. 2023; Carillo et al. 2020). This study showed that treatment with lycopene (Lyc) and L‐carnitine (L‐Car) improved liver fibrosis brought on by CCl4. Animal liver fibrosis caused by CCl4 is used as a model to mimic the pathophysiology of human liver fibrosis, and oral treatment of Lyc and L‐Car reduced the inflammatory cell infiltration caused by CCl4 (Forbes and Newsome 2016). The development of fibrosis and pro‐fibrotic cytokines is largely dependent on the activation of hepatic stellate cells and the excessive accumulation of extracellular matrix in liver tissue (Wang et al. 2021).

The assessment of the in vivo hepatoprotective effects of Lyc, L‐Car and Lyc + L‐Car was considered pertinent. Reports indicate that hepatic cytochrome P450 2E1 reductively dehalogenates CCl4, resulting in the formation of the highly reactive trichloromethyl radical (CCl3), which initiates hepatotoxic effects. Exposure of oxygen to CCl3 results in the formation of a trichloromethyl peroxyl radical (.OOCCl3). CCl4 metabolites induce hepatic oxidative stress, resulting in significant mitochondrial and nuclear damage, hepatic fibrosis, and necrotic cell death (Lee et al. 2019). Lipid peroxidation (LPO) initiates when CCl3 and OOCCl3 extract hydrogen atoms from polyunsaturated fatty acids within the cell membrane (Jiménez‐Torres et al. 2021). The significant decrease in hepatic antioxidant levels and the increase in lipid peroxidation indices corroborate prior research and elucidate the mechanism by which CCl4‐treated rats experienced hepatotoxicity (Molehin et al. 2017; Hsouna et al. 2019). Enzymatic antioxidants, such as SOD and CAT, are employed to maintain cellular oxidative stress in conditions characterized by diminished antioxidant levels and elevated lipid peroxidation (Figure 2).

Current study results for Lyc + L‐Car supplementation provided significant protection against CCl4‐induced liver fibrosis by reducing the hepatic marker enzyme levels: ALP (Figure 1A), AST (Figure 1B), ALT (Figure 1C), and bilirubin (Figure 1B) from cells into the bloodstream. An elevation in plasma levels of these enzymes is a reliable marker for assessing hepatotoxicity (Frank et al. 2020). Post CCl4 treatment, significant elevated levels of plasma ALT, AST, ALP, and bilirubin (Figure 1). The increase in alkaline phosphatase (ALP) synthesis under conditions of elevated biliary pressure and the subsequent rise in bilirubin may explain the elevated plasma levels of ALP observed in CCl4‐treated rats. However, these changes were significantly mitigated by treatment with Lyc and L‐Car, indicating their potential role in membrane stabilization. According to a recent study, hepatocyte necrosis and/or altered membrane permeability may be the cause of liver injury, as evidenced by the injury marker LDH (Bedoui et al. 2023). In the present study, the combination of Lyc and L‐Car significantly reduced the elevated LDH levels and protected the liver from damage.

Significant molecular synergy has been seen when Lyc and L‐Car are used together, which is novel because earlier research on these compounds' monotherapies for liver fibrosis has been documented (Huang et al. 2022; Li et al. 2023; Tesic Rajkovic et al. 2025; Karabulut et al. 2021; Nimbalkar and Vyawahare 2021). The ability of this novel combination (Lyc + L‐Car) to control the TGF‐β/Smad signaling cascade may account for its suppressive effect on the expression of the pro‐fibrogenic markers of TIMP‐1 and Col1α1. One of the potent inhibitors of TGF‐β1 expression is the carotenoid lycopene, which also halts Smad protein phosphorylation and the activation of hepatic stellate cells (HSCs) (Wang, Ping, et al. 2024; Mu et al. 2018). Simultaneously, L‐carnitine may enhance mitochondrial metabolism and beta‐oxidation efficiency, reducing lipotoxic stimuli that initiate fibrotic signaling. Additionally, a significant inhibition of the NF‐κB pathway is indicated by the sudden decrease in LDH levels and the restoration of SOD and CAT activities (Mollica et al. 2020). By neutralizing reactive oxygen species (ROS) and stabilizing the mitochondrial membranes, the Lyc + L‐Car treatment helps to prevent the nuclear translocation of NF‐κB, halting the progression from oxidative stress to irreversible structural destruction.

Furthermore, tissue inhibitor of matrix metalloproteinases (TIMPs) are important regulators of matrix protein formation and degradation in a healthy liver (Zhang et al. 2022). However, the activated HSCs in the liver treated with CCl4 boost TIMP‐1 secretion (Huang et al. 2024). As predicted, Lyc + L‐Car treatment decreased the level of Col1α1, however the current study's results also revealed increased expressions of Col1α1 in the livers of the CCl4 group (Figure 4; Table 1). Rats given CCl4 also had notable intracellular lipid buildup, ballooning hepatocyte degeneration, infiltration of inflammatory cells, and hepatocyte apoptosis, according to histological investigation, which supported these findings (Yang et al. 2019). Additionally, CCl4 groups that received further treatment with Lyc, L‐Car, and combined Lyc + L‐Car showed similar hepatocyte structural changes to the control group, but the Lyc + L‐Car group also shown a hopeful improvement in the fibrotic liver (Figures 5 and 6).

## 5 Conclusion

In conclusion, this in vivo study shows how well Lyc and L‐car in combination protect the liver from damage caused by CCl4. Both compounds had significant protective effects, evidenced by preserved hepatic histology, reduced oxidative stress, controlling liver enzymes balance and enhanced indices of liver function. This shows that their anti‐inflammatory and antioxidant effects worked together to make them stronger, leading to a more pronounced restoration of liver tissue integrity and normalization of biochemical indicators. The findings suggest that Lyc and L‐car may serve as a viable approach to mitigate chemically induced hepatic dysfunction, particularly when administered in conjunction. To validate these benefits and establish safe and effective dose regimens for potential therapeutic use, more study, including clinical trials, is recommended.

## Author Contributions

The listed authors have contributed equally to the work. All authors have read and agreed to publish the current version of the manuscript.

## Funding

The authors have nothing to report.

## Disclosure

The authors have nothing to report.

## Ethics Statement

Departmental Research Ethical Committee of The University of Lahore authorized the procedures used, guaranteeing adherence to animal care regulations with Reference no (UOL/DERC/22/08/7524).

## Consent

This study did not involve humans.

## Conflicts of Interest

The authors declare no conflicts of interest.

## Data Availability

The data supporting this study's findings are available from the corresponding author upon reasonable request.
